# Research on Rice Yield Prediction Model Based on Deep Learning

**DOI:** 10.1155/2022/1922561

**Published:** 2022-04-26

**Authors:** Xiao Han, Fangbiao Liu, Xiaoliang He, Fenglou Ling

**Affiliations:** ^1^College of Agriculture, Jilin Agricultural University, Changchun 130000, Jilin, China; ^2^Jilin Danong Seed Co, Ltd, Changchun 130000, Jilin, China

## Abstract

Food is the paramount necessity of the people. With the progress of society and the improvement of social welfare system, the living standards of people all over the world are constantly improving. The development of medical industry improves people's health level constantly, and the world population is constantly climbing to a new peak. With the continuous development of deep learning in recent years, its advantages are constantly displayed, especially in the aspect of image recognition and processing, it drives into the distance. Thanks to the superiority of deep learning in image processing, the combination of remote sensing images and deep learning has attracted more attention. To simulate the four key factors of rice yield, this article tries a regression model with a combination of various characteristic independent variables. In this article, the selection of the best linear and nonlinear regression models is discussed, the prediction performance and significance of each regression model are analyzed, and some thoughts are given on estimation of actual rice yield.

## 1. Introduction

Agriculture is a primary industry, and it is also a security industry [1] for China's economic construction and social development. As one of the most important kinds of grain crops, the research of rice plays an important role in agricultural production and practice [2, 3]. Especially in China, the average planting area, yield per unit area, and total yield of rice rank second in the national grain crop. As the largest rice producer and consumer in the world, it is particularly important to ensure the high yield of rice in China.

Constantly breeding new rice varieties with high yield, good stress resistance, and high nutrient utilization rate, increasing rice yield per unit area, and developing the genetic potential of rice yield as much as possible have become important goals in the field of rice breeding and cultivation in the new era [4]. Studying the characteristics of rice yield is very important for promoting land scale management, ensuring national food security, increasing farmers' income, which is of great significance to effectively alleviate the problem of food shortage [5, 6].

The traditional method of measuring rice yield in the field is destructive, that is, according to the principle of equal area or average sampling in groups, select some small fields, thresh, dry, clean, and weigh the rice after harvest, then measure the water content with moisture meter, and calculate the final rice yield according to the proportion of indica rice and japonica rice of 13.5% and 14.5% [7]. This method is not only cumbersome to operate but also needs to consume a lot of manpower and material resources. Moreover, these steps will lead to larger measurement error. In recent years, the demand for workers in China has been rising, but the cost of employing people has not decreased, but greatly increased. In some regions, the phenomenon of “cannot afford to hire” has even appeared [8].; therefore, there is an urgent need to study a new method of accurate rice yield estimation in the field. At present, governments of various countries are very concerned about food security and food shortage. Accurate estimation of crop yield is an important basis for agricultural departments at all levels to carry out cultivation management and scientific production regulation which is an important reference for countries to formulate corresponding schemes of crop management.

## 2. Deep Learning Algorithm and Its Application in Crop Yield Prediction

### 2.1. Research of Deep Learning Based Image Segmentation Algorithm

In recent years, artificial intelligence and image segmentation algorithms are developing rapidly and gradually replace the traditional method with its flexibility of self-adaptive learning from numerous samples [9]. The landmark network structure in the field of deep learning is convolutional neural network, which reduces the number of parameters and improves the generalization ability through local perception and weight-sharing [10]. The main operation of the classical convolutional neural network is to obtain the classification feature vector [11–13] by using the fully connected layer and softmax output after many convolutions. Among them, the full convolution neural network uses deconvolution to restore the size of feature map, which can not only retain the input spatial information but also obtain the output with the same size. This operation can realize the pixel-level segmentation of the image, thereby solving the problem of segmentation [14].

The differences between neural network structures become larger with the increase of network layers. Related researchers have explored different network structures [15–17] and put forward a variety of networks for image segmentation after the appearance of fully convolutional neural networks. It is mainly divided into encoding and decoding structure and expansion of convolution structure; The network representatives of encoding and decoding structure include U-net [18], Seg Net [19], Refinet [20], etc., where an encoder is used to extract image features and dimension reduction, and a decoder is used to recover image dimension and spatial information. The representative networks of expansive convolution are Deep Labv1 [21], V2 [22], V3 [23], V3+ [24],and PSPNet [25] which can increase the size of the input image even if no pooling layer is used so that each convolution can contain more information when outputting. In addition, the networks with good effect in the field of target detection have also been applied to the field of instance segmentation, and achieved good segmentation results, such as regional convolution network (R–CNN) [26], FAST R–CNN [27], Faster R–CNN [28], Maskr-CNN [29], and so on. On the basis of R–CNN, Hybrid Task Cascade (HTC) framework was proposed, which broke through the previous segmentation effect once again. In addition, many researchers have also proposed attention mechanism and applied it to segmentation networks. On this basis, some scholars put forward the DANet, which attached two attention modules to FCN and achieved the latest achievements [30]. In addition to the abovementioned networks, among the image segmentation networks, the networks for feature segmentation are constantly developing which achieved promising results in image classification and target detection. In this process, some classic network structures emerged, such as Lenet in 1998, Alex Net in 2012, Google Net and VGG in 2014, and Res Nein in 2015 [31]. With the development of technology, the complexity of the model increases, and the application fields are more extensive. Aiming at a deep learning algorithm in the field of image segmentation, Minaee and others systematically summarized and introduced all details of it, which is helpful for us to better understand and use it.

### 2.2. Production Forecast Method

Deep learning is a new technology of image processing and data analysis, which has a good effect and a great potential. With the successful application of deep learning in various fields, the prospect of smart agriculture supported by deep learning is very clear. At present, more than 40 studies in the agricultural field have adopted deep learning technology. These studies show that deep learning provides high precision, which is superior to the existing common image processing technology. In recent years, the exponential growth of remote sensing data has also provided a large number of data sources for geoscience tasks, which give full play to the role of the combination of remote sensing and deep learning in practical applications.

Convolutional neural network (CNN) is one of the most successful deep learning frameworks, which greatly reduces the training parameters and improves the computational efficiency and generalization ability. Recently, many scholars have made a lot of attempts based on CNN structure and applied them to their respective research fields to identify different types of targets in satellite and aerial images through innovative algorithms. Landsat series satellites are widely used data sources, with a spatial resolution of 30 m and a temporal resolution of 16 days. In 2013, China launched the GF-1 satellite, which is equipped with two full-color cameras with a resolution of 2 m and a multispectral camera with a resolution of 16 m. The revisit time of GF-1 satellite is about 4 days. Considering its spatial and temporal resolution, it has obvious advantages. GF-1 is a high-resolution remote sensing image, which contains more spatial information than the medium-resolution remote sensing image. According to this feature, more detailed field crop feature information can be extracted to achieve the purpose of precision in agriculture. Thus far, there is little research on the application of GF-1 satellite images to farmland extraction, especially the advanced deep learning technology.

Prediction model based on image feature is one of the important methods of deep learning in yield prediction of agricultural product. The output can be divided into two types, loss and lossless in vivo prediction. The loss prediction is to measure the length, width, and weight of the ear based on image processing technology after harvesting the mature rice ear, and the prediction model can be established by extracting grain yield characters such as grain length, grain width, total number of grains, aspect ratio, standard deviation, and 1000-grain weight. Some researchers have designed a set of automatic threshing, image acquisition, extraction of grain length, grain width and other data, and automatic bagging. By this device, grain characteristics can be automatically extracted, which is highly correlated with yield and can be used for damage prediction [32]. Lossless yield prediction is mainly based on panicle cutting, color feature extraction, and regression model with yield through RGB images. A small area of wheat is extracted from the field, RGB images of wheat are taken with the background board, the panicles are segmented by color space conversion and image processing technology, the number of ears is identified, and the number of grains is predicted. Establishing a model to predict the wheat yield per unit area means by employing the image of a single ear and MATLAB image processing, the texture features of some ear images are extracted, and the parameters significantly related to panicle yield are selected, and the prediction model is established by multiple linear regression. UAV takes RGB pictures of rice canopy, uses K-means clustering to segment the pictures, extracts rice ears, and obtains the number of ears which forecast the output.

## 3. Experimental Analysis of Rice Yield Prediction Based on Deep Learning

### 3.1. Overall Design

In this experiment, a total of 207 paddy plots in the field were measured. Each plot planted 20 rice plants of the same variety, but the rice varieties in different plots were different. Each rice plot separately extracts the image features of the rice ear plot, the detailed image features of a single rice ear and the seed test features of a single grain. Then, the regression equation between image features and plot total output is constructed, so as to realize the purpose of rice plot yield prediction.

Regression analysis is a method to build a complex regression equation based on the analysis of the correlation between the independent variables of image features and the dependent variables of plot output. According to the difference in the number of independent variables, regression analysis can be divided into univariate regression and multivariate regression. The so-called “univariate correlation regression prediction” is to construct correlation analysis between an independent variable and a dependent variable. Multivariate regression prediction means that multiple independent variables are integrated to predict dependent variables.

Usually, the construction of regression model includes the following steps:

1. Make a scatter chart for each independent variable, observe the change trend of independent variable, and analyze whether the dependent variable conforms to the normal distribution, etc., and investigate whether it can be constructed by linear model.
2. Selection of characteristic variables and construction of regression model.
3. Make a scatter plot between the predicted regression value and the true value, and test the regression equation. The main test contents include goodness of fit test, *F*-test for the significance of regression equation, and *T*-test for the significance of regression parameters.
4. Residual analysis and collinearity diagnosis of regression results, including whether the residual distribution of the main accords with the normal distribution, the judgment of multicollinearity of the regression equation, etc.
5. Analysis and diagnosis of regression characteristics in regression model, explanation and discussion of prediction model.

### 3.2. The Normality Test of the Total Output of the Plot

In the actual research on the estimation of total yield of rice plot, it is necessary to measure the normal distribution characteristics of dependent variables (total yield of plot) at first. The common measurement method is to calculate the skewness according to the histogram of dependent variable to judge the normality of the histogram. Skewness is a measure of skewness of histogram distribution of target data. For a set of data, the histogram distribution is not necessarily symmetrical, and it may be skewed from left to right. Usually, the mode of the histogram is located on the left side of arithmetic mean, which is called left deviation. At this time, the calculated skewness is positive. When the mode of histogram is on the right side of arithmetic mean, the whole distribution is in a state of right deviation, and the skewness is negative. Therefore, when the skewness is closer to 0, it means that the histogram is closer to normal distribution.

**Figure 1:**
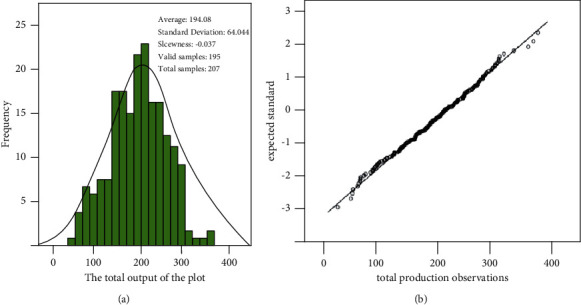
Histogram and Q-Q diagram of total production in the field.

The histogram and Q-Q diagram of the total yield (dependent variable) of rice plot are shown in Figure 1.

From the statistical description of the histogram in Figure 1(a), the skewness of the histogram is −0.037, which is close to 0, indicating that the normality of the dependent variable is better. The Q-Q diagram of the dependent variable (as shown in Figure 1(b)) further verifies the normal property of the distribution. The closer the distribution of observation points in Figure 1(b) is to a straight line, the better the normal property of its distribution.

The normality of dependent variables is tested by using two testing methods in SPSS as shown in Table 1. In this article, the number of effective rice plots is 179 that is not large, so the Kolmogorov–Smirnov normal test results shall prevail. It can be seen from Table 1 that the significant P of the dependent variable is 0.200, which is higher than the threshold of 0.05. The above inspection results show that it is significant that the total yield of rice plot obeys the normal distribution, so the linear model can be used to estimate the yield.

### 3.3. Total Output Estimation of Four Factors Related to Simulated Rice Yield

Four key factors are related to rice yield, including number of ears per unit area, number of grains per ear, seed setting rate, and 1000-grain weight. In this article, based on the simulation of four factors related to rice yield, the characteristics with the ability are selected to represent the yield to establish a regression model and estimate the yield of rice plot.

The corrected area of rice ears in different angles can reflect the number of ears per unit area of rice to a certain extent. However, the area and ear length of detail image about a single rice ear are related to the number of grains per ear. For each plot, because the varieties of rice in the plot are consistent, the characteristics of grain seed test of single rice, for example, the seed setting rate and 1000-grain weight can represent the seed setting rate and 1000-grain weight of the whole plot to a certain extent. Therefore, the key point of this section is to build a regression model by the corrected area of the image taken from different angles, the corrected area of detail image about a single rice ear, the ear length, and the seed test parameters of the single rice grain, so as to realize the estimation of the yield. For the convenience of description, we stipulate that the symbol TPCA is used to represent the corrected area of the bottom-view of the rice ear plot, the symbol OPCA is used to represent the corrected area of the top-view of the rice ear plot, the symbol SPCA is used to represent the corrected area of detail image about a single rice ear, the symbol SPL is used to represent the ear length of detail image about a single rice ear, the symbol SRSR is used to represent the grain setting rate of a single rice, and the symbol “SRTW” indicates the 1000-grain weight of rice grains per plant.

The purpose of regression is to obtain the target value of numerical data according to the empirical value. Mathematically speaking, regression is to calculate a regression equation so that the predicted output can be obtained for each input. The goodness-of-fit (*R*^2^) is often used to evaluate the results of regression, and its calculation expression is shown in formula (1):

(1) $$\begin{array}{c} R^{2}=1-\frac{\text{SSE}}{\text{SST}}. \end{array}$$

Among them, SSE represents the sum of squares of sample residuals, also known as 2*L* normal form; whereas SST is the sum of the total squares of samples, and the calculation expressions of SSE and SST are shown in formulas (2) and (3):

(2) $$\begin{array}{c} \text{SSE}=\sum_{i=1}^{N}\left(y_{i}-y_{i}^{*}\right), \end{array}$$

(3) $$\begin{array}{c} \text{SST}=\sum_{i=1}^{N}\left(y_{i}-\bar{y}\right). \end{array}$$

where *y*_*i*_^*∗*^ indicates the prediction of the *i*th sample, *y*_*i*_ represents the true result of the *i*th sample, and $\bar{y}$ is the average of all true values of the sample. Actually, when the number of independent variables in the model increases, the *R*^2^ of regression fitting will also change, so the number of independent variables should be considered when analyzing the *R*^2^ of regression model. In SPSS, the adjusted goodness-of-fit (adjusted *R*^2^) is generally used to characterize the fitting results after comprehensive investigation of independent variable degrees of freedom. The expression of adjusted *R*^2^ is shown in formula (4):

(4) $$\begin{array}{c} \text{Adjusted }R^{2}=1-\frac{N-1}{N-M-1}\left(1-R^{2}\right), \end{array}$$

where *N* represents the total number of samples, and *M* represents the degree of freedom of independent variables (i.e., the number of sample independent variables).

#### 3.3.1. Yield Prediction Model Based on Rice Ear Area

The area of images taken from different angles can reflect the number of ears per unit area to a certain extent, and there must be a certain correlation between it and rice yield. Therefore, this section mainly studies the regression analysis between TPCA, OPCA, and field plot rice yield (FPRY).

**Figure 2:**
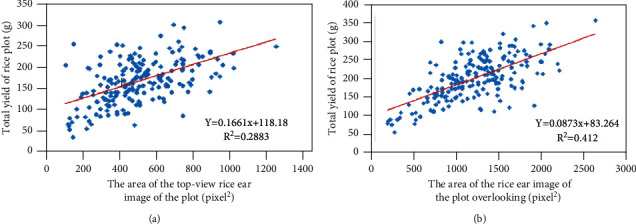
Correlation analysis between the TPCA, OPCA, and FPRY.

The scatter diagram between the corrected area of plot rice spike image and plot rice yield is shown in Figure 2. In Figure 2(a), the goodness-of-fit *R*^2^ between TPCA and FPRY is 0.2883, whereas the goodness-of-fit *R*^2^ between OPCA and FPRY can reach 0.412, as shown in Figure 2(b). This shows that the corrected area of rice ear image in plot is a significant yield prediction feature.

Figure 2 shows the correlation analysis of univariate linear model and further carries out the prediction of rice yield based on univariate nonlinear model. Among them, the regression goodness-of-fit *R*^2^ of the index model for predicting rice yield, top view and bottom view is 0.2987 and 0.4136. The regression goodness-of-fit *R*^2^ of the numeric model for predicting rice yield from top to bottom is 0.2937 and 0.4389. The regression goodness-of-fit *R*^2^ of the cubic polynomial model for predicting the rice yield under the top and bottom conditions is 0.3015 and 0.4524. The power function model is used to predict the rice yield and the regression goodness-of-fit *R*^2^ of the top and the bottom views is 0.3487 and 0.5133.

Table 2 shows the goodness-of-fit *R*^2^, adjusted *R*^2^, *F*-test, and model significance test of linear and nonlinear univariate regression prediction in the form of data tables. Through the analysis of Table 2, it shows that the optimal univariate regression model of TPCA, OPCA, and FPRY adopts the form of power function. In this form, the goodness-of-fit *R*^2^ of TPCA and FPRY can reach 0.345, while the goodness-of-fit *R*^2^ of OPCA and FPRY is 0.511. The relationship between the corrected rice ear area and the total yield in this plot should conform to the structure of power function.

If TPCA and OPCA are used as the input independent variables of the regression model (Model 1), and the regression equation is constructed by linear model, as shown in Table 3, then the adjusted *R*^2^ of model 1 is only 0.385, which is lower than the regression prediction *R*^2^ = 0.412 of single variable OPCA and FPRY. After the introduction of TPCA, FPRY's results decreased, which indicated that OPCA was more reasonable than TPCA in predicting rice plot yield.

#### 3.3.2. Yield Prediction Model Based on the Characteristics of Rice Ear Area and Single Ear Detail Image

The features extracted from the detailed image of a single rice ear mainly include two features, namely the single spike corrected area (SPCA) and the single spike length (SPL). These two characteristics are related to the number of grains per panicle among the four factors of rice yield to some extent, so SPCA and SPL also have guiding significance for the prediction of FPRY.

Pearson correlation coefficient and significance between SPCA and SPL and FPRY are shown in Table 4. Pearson correlation coefficient shows that the correlations between SPCA, SPL, and FPRY are 0.470 and 0.376, respectively. The results show that there is a certain correlation between SPCA and SPL, and FPRY. If these two variables are added to Model 1 as input features, Model 2 consisting of four independent variables will be formed. According to linear regression analysis, it can be seen from Table 5 that the goodness-of-fit *R*^2^ of model 2 is 0.413. The goodness-of-fit *R*^2^ of comparison model 1 is 0.385, and the results show that the introduction of detailed features of a single panicle can improve the predictive ability of FPRY.

#### 3.3.3. Yield Prediction Model Based on Rice Ear Area, Detailed Image Features, and Single Seed Test Characters

Among the seed test characters of a single plant, the most important are the two characteristics of grain setting rate and 1000-grain weight. As the varieties of 20 rice plants in the same plot are the same, the seed setting rate and 1000-grain weight of each plant can reflect the seed setting rate and 1000-grain weight of the whole plot to a certain extent.

Table 6 shows the introduction of two characteristics of single rice setting rate (SRSR) and single rice 1000-grain weight (SRTW) into Model 2, and the fitting of regression model (Model 3) is constructed with six variables. It can be seen from Table 6 that the adjusted goodness-of-fit *R*^2^ of Model 3 is 0.456. The goodness-of-fit *r*^2^ of comparative model 2 is 0.413. The results showed that the introduction of single seed test traits could improve the predictive ability of FPRY.

#### 3.3.4. Screen of Characteristic Traits Based on Stepwise Linear Regression

There are two methods to screen common characteristic variables, one is to use all subsets regression, the other is to use stepwise linear regression. In this article, stepwise linear regression is used to screen characteristic variables with characterization ability. In the stepwise linear regression, first, the characteristic variables with the highest correlation are screened, and then new variables are introduced one by one. While every time a variable is introduced, *F-*test and significance *T-*test of the selected characteristic variables should be carried out. Specifically, assuming that the number of characteristic variables is *n*, the independent variables of the regression equation can be expressed as *x*_1_, *x*_2_, *x*_3_,…, *x*_*n*_, for dependent variable *y*, the regression expression is shown in formula (5):

(5) $$\begin{array}{c} Y=\alpha +\alpha_{1}x_{1}+a_{2}x_{2}+\cdots +a_{i}x_{i}+\cdots \alpha_{n}x_{n}, \end{array}$$

where *i*=1,2,3,…, *na*_*i*_ is a regression coefficient of independent variable *x*_*i*_, assuming the maximum value of *F*-test statistic of *a*_*i*_ is *F*_*a*_*i*__, then for the significance level of 0.05, suppose that *F*_*a*_*i*__ is greater than or equal to the critical threshold, so *x*_*i*_, the corresponding characteristic independent variable of *a*_*i*_ can be introduced. Repeat the above process of introducing characteristic variables, but after each introduction of variables, it is necessary to carry out significance *T*-test on the selected characteristic variables, which is used to ensure that all the selected characteristic variables are significant. If a characteristic variable is introduced, the previously introduced variable is not significant, then the characteristic variable should be eliminated, and finally repeat this process until no new variables can be introduced.

The main advantage of stepwise linear regression is that while constructing regression equation, it can also realize the screening of characteristic variables, which is convenient for people to understand existing models and make corresponding changes. Among them, the first characteristic variable screened out has the highest importance, followed by the second, and so on. After finding the appropriate number of feature variables, the collection of unimportant features can be stopped.

**Figure 3:**
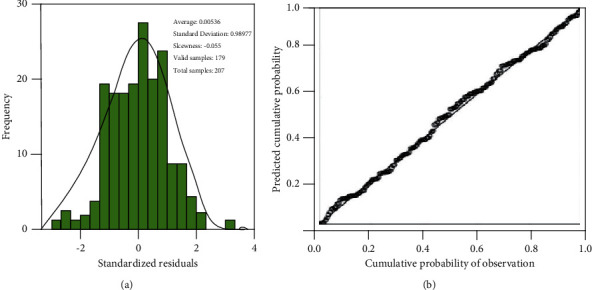
Prediction of the residual distribution histogram and P–P graph for Model 4.

The results of stepwise linear regression after inputting six characteristic parameters (TPCA, OPCA, SPCA, SPL, SRSR, and SRTW) are shown in Table 7. After all six parameters were entered, three variables were screened out, among which OPCA was the first variable screened out, then SRSR, and finally SPCA. That is to say, all these three parameters have passed the *T*-test of the model, and all of them have strong ability in yield characterization. In Table 7, the adjusted goodness-of-fit *R*_2_ of the yield regression model can reach 0.453, which is called Model 4 in this article. Model 4 consists of three independent variables, namely OPCA, SRSR, and SPCA. Compared with the regression Model 3 consisting of six independent variables, the adjusted *R*_2_ of Model 4 only dropped from 0.456 to 0.453. The above results show that among the six characteristic variables selected by the four factors of simulated yield, the relationship among OPCA, SRSR, SPCA, and FPRY is closer, so the following regression analysis focuses only on these three selected characteristic variables. The residual *n* histogram and P–P diagram according to Model 4 are shown in Figure 3.

In Figure 3(a), the histogram skewness of residual distribution in FPRY's prediction is −0.055, which is close to 0. At the same time, from the P–P diagram of cumulative probability distribution (Figure 3(b)), it can seen that the observed values are all distributed on the diagonal line, and these results show that it is feasible to use this linear Model 4 to predict FPRY.

## 4. Conclusion

With the increase of population and the continuous improvement of people's living standards, the demand for food is also increasing. As the main food crop in China, rice has always been the main research object of breeders, and yield prediction has always been an important research orientation of rice. The research structure of this article shows that with a single independent variable, the correlation between OPCA and the total output of rice plot is much higher than that between TPCA and the total output of rice plot. The accurate segmentation of rice ears is of great significance to the accurate estimation of yield, and the image taken from the perspective of overlooking plays a more obvious role in the estimation of yield.

## Figures and Tables

**Table 1:** Results of the normality test for rice yield in field plot.

	Kolmogorov–Smirnov			Shapiro–Wilk		
	Statistics	Df	Salience	Statis	Df	Salience
Total plot yield (dependent variable)	0.30	195	0.200b	0.995	195	0.715

**Table 2:** Parametric predictive model analysis of the single variables.

	Univariate predictive models	*R* ^2^	Adjusted *R*^2^	Standard estimate error	*F* Test	Salience
TPCA	Linear	0.288	0.285	53.353	76.549	0.000
	Index	0.299	0.295	0.317	S0.4S6	0.000
	Logarithm	0.294	0.290	53.148	78.600	0.000
	Polynomial	0.301	0.290	53.137	26.904	0.000
	Power function	0.349	0.345	0.306	101.171	0.000

OPCA	Linear	0.412	0.409	49.298	134.519	0.000
	Index	0.414	0.411	0.299	135.426	0.000
	Logarithm	0.439	0.436	48.156	150.185	0.000
	Polynomial	0.452	0.444	47.823	52.324	0.000
	Power function	0.513	0.511	0.272	202.493	0.000

**Table 3:** Two-variable regression: Model 1.

	*R* ^2^	Adjusted *R*^2^	Significance of the *F* value	Durbin–Watson
Model 1	0.391	0.385	0.000	1.696

**Table 4:** Correlation between single panicle detail image traits and plot yields.

	Pearson correlation coefficient (R)	Correlation coefficient dominance
Single Inaho image correction surface (SPCA)	0.470	0.000
Single ear length (SPL)	0.376	0.000

**Table 5:** Plot rice yield regression: Model 2.

	*R* ^2^	Adjusted *R*^2^	Significance of the *F* value	Durbin–Watson
Model 2	0.425	0.413	0.000	1.699

**Table 6:** Plot rice yield regression: Model 3.

	*R* ^2^	Adjusted *R*^2^	Significance of the *F* value	Durbin–Watson
Model 3	0.475	0.456	0.000	1.895

**Table 7:** Filtering traits using stepwise linear regression.

	*R* ^2^	Adjusted *R*^2^	Significance of the *F* value	Durbin–Watson
Number of rounds 1a	0.380	0.376	0.000	
Number of rounds 2b	0.425	0.419	0.000	1.790
Number of rounds 3c	0.462	0.453	0.001	
(Model 4)				

a, predicted value: (constant), OPCA, b, predicted value: (constant), OPCA, SRSR, c, predicted value: (constant), OPCA, SRSR, SPCA.

## Data Availability

The dataset can be accessed upon request.
